# Data on a real-time tripodal colorimetric/fluorescence sensor for multiple target metal ions

**DOI:** 10.1016/j.dib.2018.06.096

**Published:** 2018-07-02

**Authors:** Rosita Diana, Ugo Caruso, Simona Concilio, Stefano Piotto, Angela Tuzi, Barbara Panunzi

**Affiliations:** a Department of Chemical Sciences, University of Napoli Federico II, via Cintia, 80126 Napoli, Italy; b Department of Agriculture, University of Napoli Federico II, via Università 100, 80055 Portici, NA, Italy; c Department of Pharmacy, University of Salerno, via Giovanni Paolo II 132, 84084 Fisciano, SA, Italy; d Department of Industrial Engineering, University of Salerno, via Giovanni Paolo II 132, 84084 Fisciano, SA, Italy

## Abstract

Currently considerable research both in life and in environmental sciences is dedicated to chemosensors able to detect metals of biological interest such as zinc and iron or other toxic and carcinogenic, as cadmium, mercury, chromium, lead. Recently, a new chemosensor strategy of “single chemosensor for multiple metals” has emerged. For this scope, many fluorescent sensors for Cd(II) and Zn(II) have been designed and synthetized, as ligand systems or in polymeric matrices [1], [2], [3]. The data presented in this article include experimental data on the of a pyridyl/phenolic/benzothiazole functionalized colorimetric receptor (**BPAP**) and its selectively recognise Fe(III) and Fe(II) ions with visible, naked eye colour changes and fluorometric selectivity towards Zn^2+^ and Cd^2+^ ions in aqueous medium.

This article is submitted as a companion paper to Caruso et al. (2018) [4].

**Specifications Table**

**Table:** 

Subject area	*Chemistry, Materials Science*
More specific subject area	*Electro-optic field sensors*
Type of data	*Crystal data and structure refinement, NMR spectrum, tables and figure*
How data was acquired	*NMR recorded in DMSO using Bruker Spectrometers operating at 400 MHz.*
	*UV-Visible and fluorescence spectra recorded with JASCO spectrometers.*
	*Single crystals X-ray structural analysis performed on a BrukerNoniusKappaCCD diffractometer equipped with Oxford Cryostream apparatus.*
Data format	*Raw data and their elaborations*
Experimental factors	*The data concerns structural information, UV/Vis calculation and some spectroscopic raw data*
Experimental features	*Elaboration of X-ray diffraction data and UV/Vis curves*
Data source location	*Naples, Italy*
Data accessibility	*Data is within this article*

## Value of the data

• The data show some molecular structure of **BPAP** along **a** and **c** axes.
• The data report relevant structural data of **BPAP** and its zinc complex (lengths and angles).
• The data report Job׳s plot analysis for the binding Zn(II) and Cd(II) with ligand system.
• ^1^H NMR and ^13^C NMR of BPAP and BPAP metal complexes are reported.

## 1 Data

The data presented in this article are related to the research article entitled “A real-time tripodal colorimetric/fluorescence sensor for multiple target metal ions” [4]. Recently an impressive progress has been done toward the design and synthesis of novel sensitive ligands and fluorescent materials [5], [6], [7], [8]. The data presented here include experimental data on the of a pyridyl/phenolic/benzothiazole functionalized colorimetric receptor (**BPAP**) and its selectively recognise Fe(III) and Fe(II) ions with visible, naked eye colour changes and fluorometric selectivity towards Zn^2+^ and Cd^2+^ ions in aqueous medium.

The following data are a necessary support for the identification of materials and properties of the ligand system and its complexes.

## 2 Experimental design, materials and methods

**Fig. 1:**
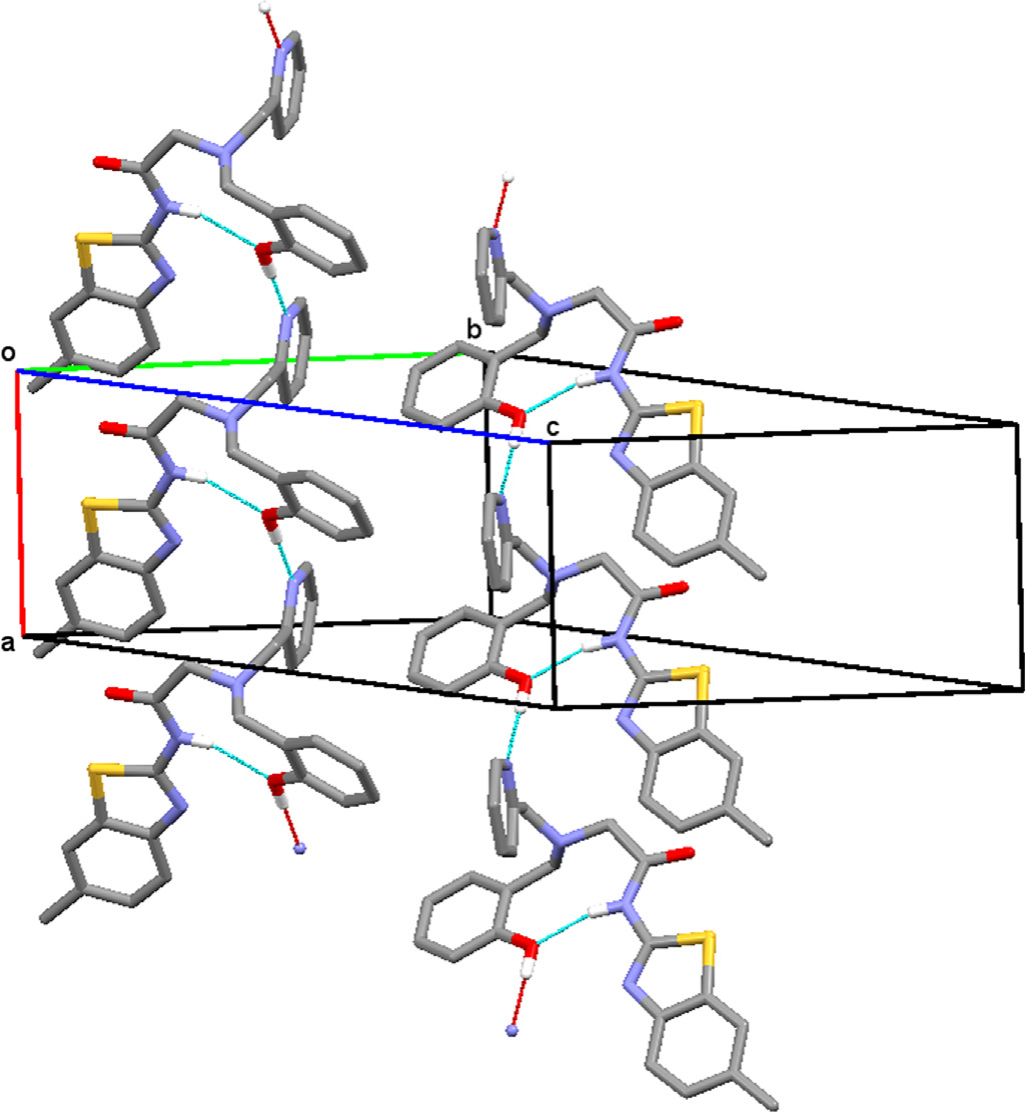
Chains of H bonded molecules of **BPAP** along **a** axis. Only the hydroxy H atom is drawn for clarity.

**Fig. 2:**
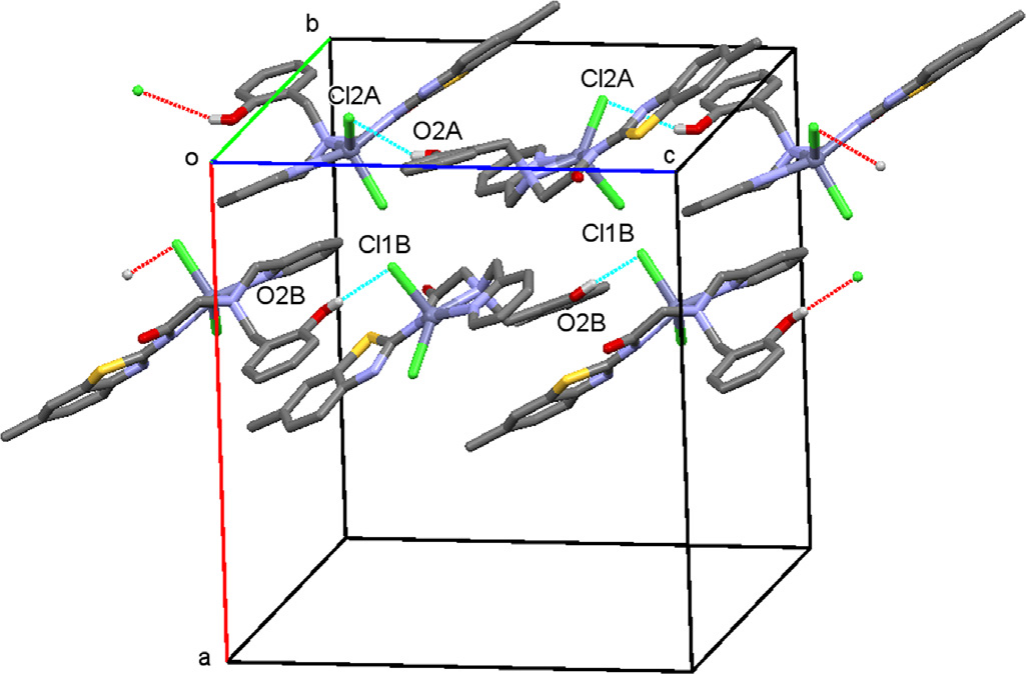
Chains of Zn-**BPAP** molecules running along **c** axis direction. Only the hydroxy H atom is drawn for clarity.

Structural analysis of single crystals of ligand and its Zinc complex has been performed on a BrukerNoniusKappaCCD diffractometer equipped with Oxford Cryostream apparatus (graphite monochromated MoK_α_ radiation, λ = 0.71073 Å, CCD rotation images, thick slices, φ and ω scans to fill asymmetric unit). Semiempirical absorption corrections (SADABS [9]) were applied. Both the two structures were solved by direct methods (SIR97 program [10]) and anisotropically refined by the full matrix least-squares method on *F*^2^ against all independent measured reflections using SHELXL-2016 [11] and WinGX software [12]. Crystal data and structure refinement details are reported in Table 1. Relevant bond lengths and angle are reported in Table 2. The figures were generated using ORTEP-3 [13] and Mercury CSD 3.9 [14] programs. Molecular structure of **BPAP** along **a** axis is shown in Fig. 1. Molecular structure of the complex Zn-**BPAP** along **c** axis is shown in Fig. 2.

**Table 1:** Structural data and refinement details for **BPAP** and Zn-**BPAP**.

	**BPAP**	**Zn-BPAP**
CCDC number	1582069	1582070
Empirical formula	C23H22N4O2S	C23H22Cl2N4 O2SZn
Formula weight	418.50	554.77
Temperature (K)	298(2)	173(2)
Wavelength (Å)	0.71073	0.71073
Crystal system (Å)	Triclinic	Monoclinic
Space group	P -1	P2_1_/c
*a* (Å)	7.2950(15)	18.542(5)
*b* (Å)	16.3670(18)	14.650(3)
*c* (Å)	18.592(2)	17.217(2)
⎕ (°)	77.506(8)	90.
⎕ (°)	87.794(11)	92.373(13)
⎕ (°)	85.826(11)	90.
Volume (Å^3^)	2160.9(6)	4672.8(17)
Z	4	8
Dcalc (Mg/m3)	1.286	1.577
⎕ (mm^−1^)	0.177	1.399
F(000)	880	2272
Crystal size (mm)	0.480 × 0.150 × 0.020	0.200 × 0.060 × 0.040
θ range (°)	2.333 to 25.996	2.602 to 27.022
Limiting indices	−8≤h≤8, −20≤k≤20, −22≤l≤22	−23≤h≤23, −18≤k≤18, −21≤l≤21
Reflections collected / unique	19,458 / 8234 [R(int) = 0.0671]	39,344 / 10,013 [R(int) = 0.1524]
Refinement method	Full-matrix least-squares on F ^2^	Full-matrix least-squares on F ^2^
Data / restraints / parameters	8234 / 0 / 556	10,013 / 0 / 609
Goodness-of-fit on F^2^	1.034	1.079
Final R indices [I>2sigma(I)]	R1 = 0.0628, wR2 = 0.1340	R1 = 0.0787, wR2 = 0.1561
R indices (all data)	R1 = 0.1416, wR2 = 0.1695	R1 = 0.1891, wR2 = 0.2013
Largest diff. peak and hole (eA^−3^)	0.448 and −0.283	0.800 and −0.878

**Table 2:** Selected bond lengths (Å) and angles (°).

	**BPAP**		**Zn-BPAP**	
	Molecule A	Molecule B	Molecule A	Molecule B
C9-O1	1.219(4)	1.212(4)	1.221(9)	1.220(9)
C9-N2	1.3578(5)	1.357(5)	1.372(9)	1.345(10)
S1-C8	1.746(3)	1.742(4)	1.724(8)	1.734(7)
S1-C6	1.734(4)	1.730(4)	1.754(7)	1.747(7)
Zn1-N2			2.207(6)	2.213(6)
Zn1-N3			2.182(6)	2.165(6)
Zn1-N4			2.157(6)	2.140(6)
Zn1-Cl1			2.250(3)	2.289(2)
Zn1-Cl2			2.288(3)	2.282(2)
C8-N2-C9	126.2(3)	126.0(3)	116.4(6)	117.0(6)
N2-C9-C10	115.8(3)	115.3(3)	113.6(6)	114.1(7)
O1-C9-N2	122.4(4)	123.2(4)	125.7(7)	126.6(7)

All crystal data were deposited at Cambridge Crystallographic Data Centre with assigned number CCDC 1582069 (**BPAP**) and 1582070 (**Zn-BPAP**). These data can be obtained free of charge from www.ccdc.cam.ac.uk/data_request/cif.

**Fig. 3:**
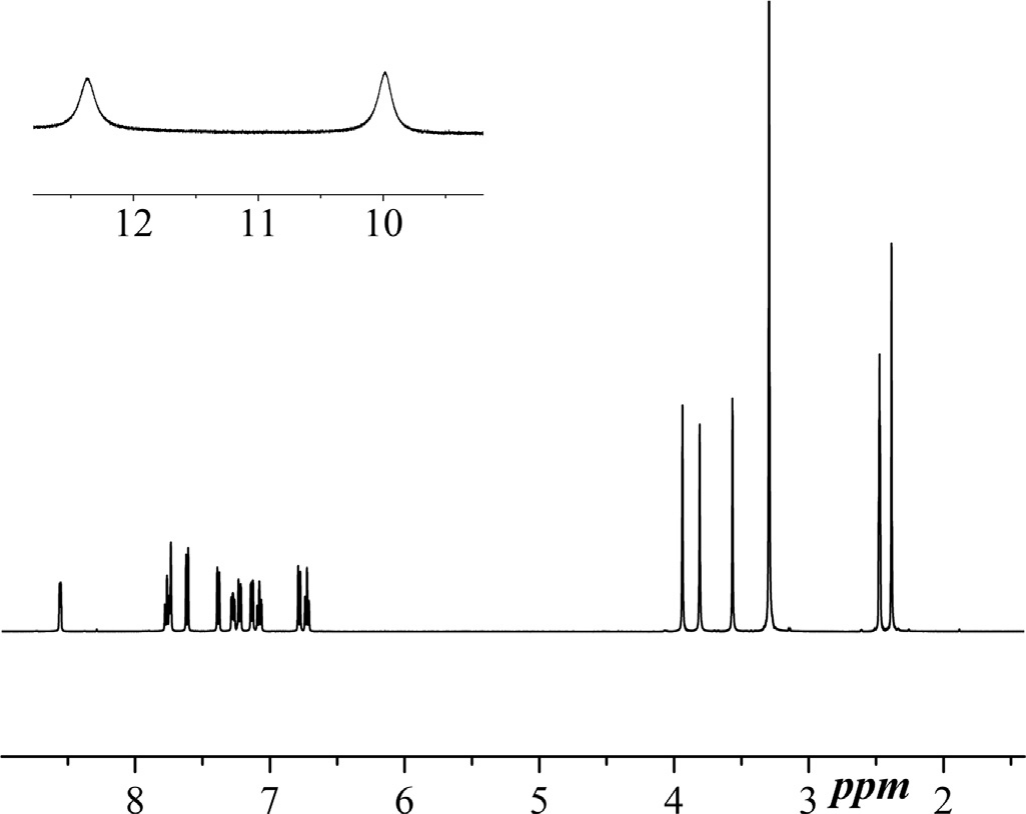
^1^H NMR spectrum of **BPAP**.

**Fig. 4:**
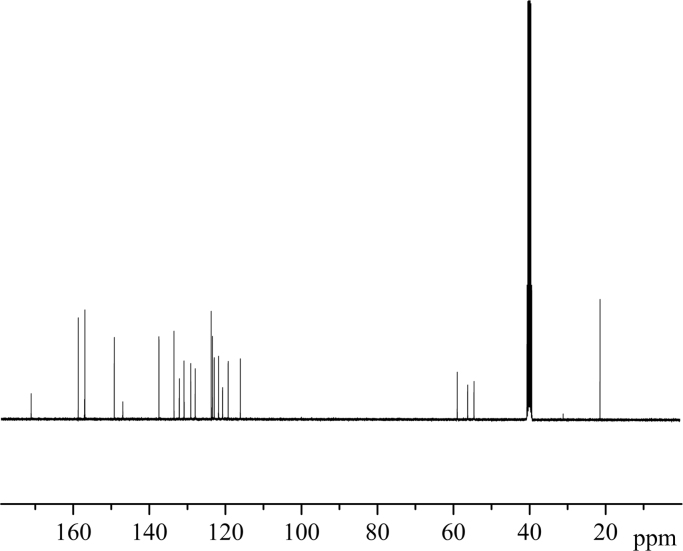
^13^C NMR spectrum of **BPAP**.

**Fig. 5:**
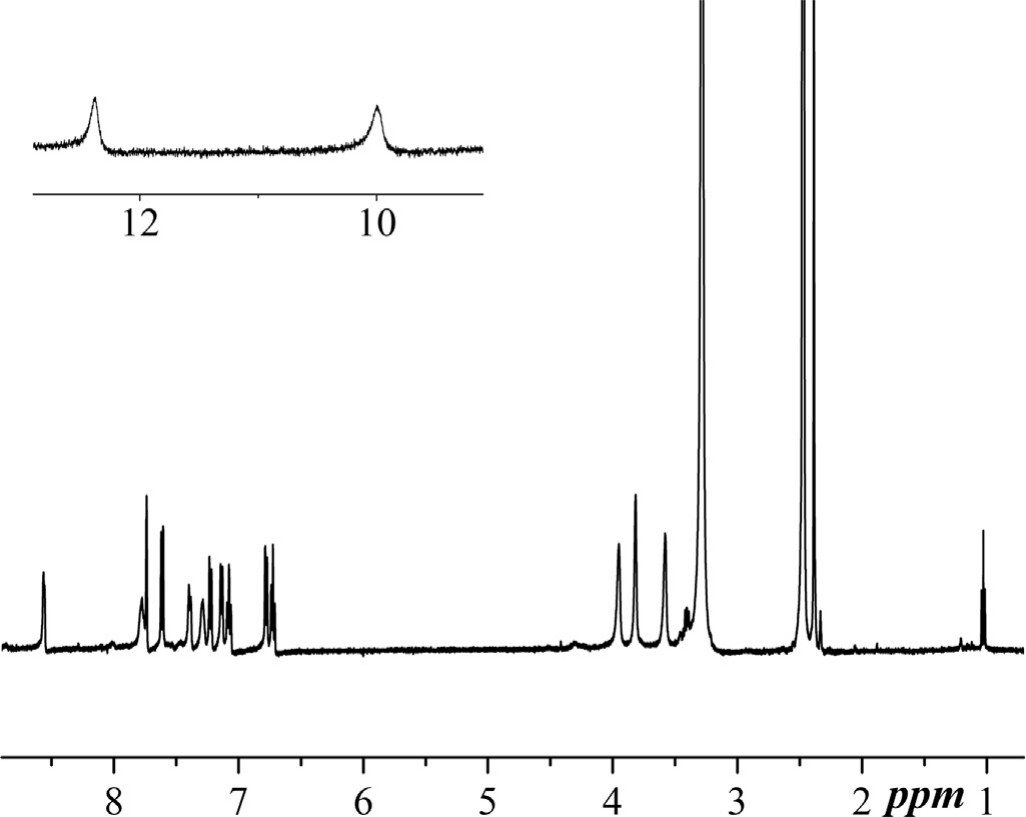
^1^H NMR spectrum of Zn-**BPAP**.

NMR spectra were recorded in DMSO using a Bruker Spectrometer operating at 400 MHz. For BPAP, both ^1^H and ^13^C NMR are reported in Fig. 3, Fig. 4. In Fig. 5, ^1^H NMR spectrum of Zn-BPAP is shown.

**Fig. 6:**
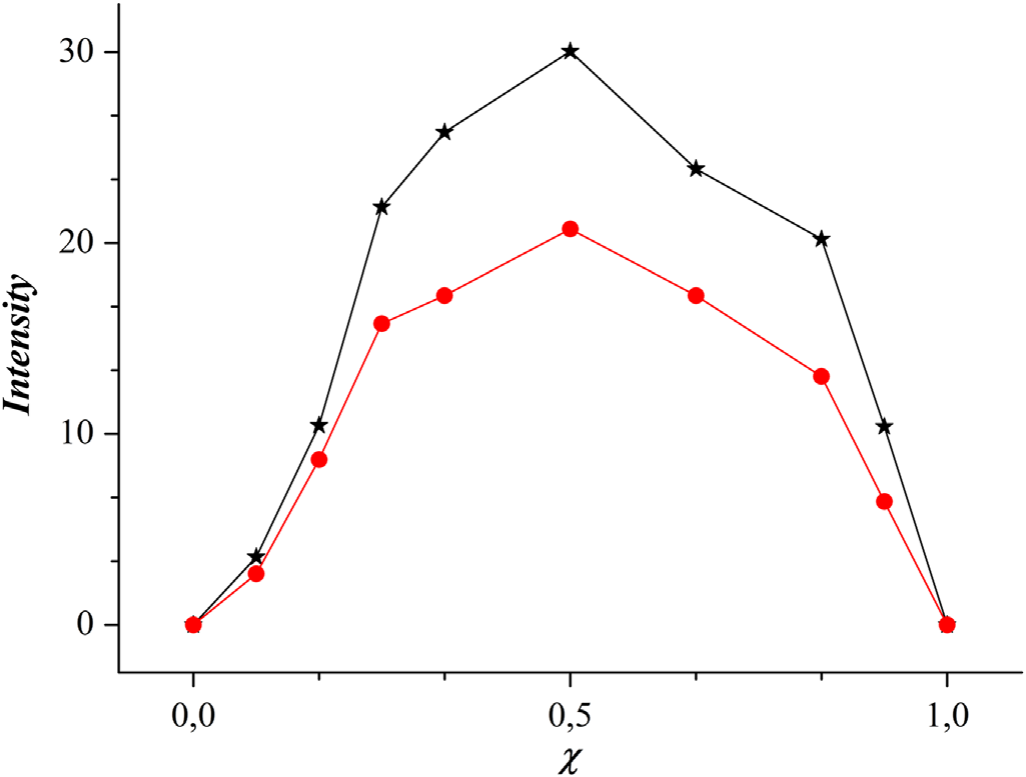
Job׳s plot analysis for the binding Zn(II) (black curve) and Cd(II) (red curve) with **BPAP**.

Job׳s plot measurement of Zn^2+^ and Cd^2+^ (Fig. 6) has been performed on 500μM solutions of Zn(II) chloride (or Cd(II) chloride) in bidistilled water (pH 6.25) and 500μM of **BPAP** in ethanol. Volumes of 3.00, 2.75, 2.50, 2.00, 1.50, 1.00, 0.50, 0.25 and 0 mL of the solution of ligand were taken and transferred to vials and volumes of 0, 0.25, 0.50, 1.00, 1.50, 2.00, 2.50, 2.75, 3.00 mL of metal ion added, each vial having a total volume of 3.0 mL. Fluorescence spectra were recorded at room temperature after shaking each vial for a few seconds.
