# Supplementary material for: Data on a real-time tripodal colorimetric/fluorescence sensor for multiple target metal ions
Source: Data Brief. 2018 Jul 2;19:2119–25. doi: 10.1016/j.dib.2018.06.096 (PMC6141418; doi:10.1016/j.dib.2018.06.096)
Supplement: Supplementary file 1 — Supplementary material [file mmc1.pdf]

Conflicts of Interest Statement

Title: Data on a real-time tripodal colorimetric/fluorescence sensor for multiple target metal ions

Authors: Rosita Diana[†], Ugo Caruso[†,*], Simona Concilio[⊥], Stefano Piotto[§], Angela Tuzi[†] and Barbara Panunzi[‡]

Affiliations:

† Department of Chemical Sciences, University of Napoli Federico II, via Cintia, 80126 Napoli, Italy
‡ Department of Agriculture, University of Napoli Federico II, via Università 100, 80055 Portici NA, Italy
§ Department of Pharmacy, University of Salerno, via Giovanni Paolo II 132, 84084 Fisciano SA, Italy
⊥ Department of Industrial Engineering, University of Salerno, via Giovanni Paolo II 132, 84084 Fisciano SA, Italy

Contact email: ugo.caruso@unina.it

The authors confirm that there are no known conflicts of interest associated with this publication and there has been no significant financial support for this work that could have influenced its outcome.

According with all authors, this declaration is signed only by the corresponding author.

Prof. Ugo Caruso
